# Feasibility of inter-hospital transportation using extra-corporeal membrane oxygenation (ECMO) support of patients affected by severe swine-flu(H1N1)-related ARDS

**DOI:** 10.1186/1757-7241-19-32

**Published:** 2011-05-27

**Authors:** Marco Ciapetti, Giovanni Cianchi, Giovanni Zagli, Cesare Greco, Andrea Pasquini, Rosario Spina, Stefano Batacchi, Manuela Bonizzoli, Massimo Bonacchi, Chiara Lazzeri, Pasquale Bernardo, Adriano Peris

**Affiliations:** 1 Anesthesia and Intensive Care Unit of Emergency Department, Careggi Teaching Hospital, Florence, Italy; 2 Postgraduate School of Anesthesia and Intensive Care, Faculty of Medicine, University of Florence, Italy; 3 Cardiac Surgery, Heart and Vessel Department, Careggi Teaching Hospital, Florence, Italy; 4 Intensive Cardiac Coronary Unit, Heart and Vessel Department, Careggi Teaching Hospital, Florence, Italy

## Abstract

**Background:**

To describe the organization of an ECMO-centre from triage by telephone to the phase of inter-hospital transportation with ECMO of patients affected by H1N1-induced ARDS, describing techniques and equipment used.

**Methods:**

From September 2009 to January 2010, 18 patients with H1N1-induced ARDS were referred to our ECMO-centre from other hospitals. Six patients had contraindications to treatment with ECMO and remained in the local hospital. Twelve patients were transported to our centre and were included in this study. Four patients were transported on ECMO (Group A) and eight on conventional ventilation (Group B). The groups were compared on the basis of adverse events during transport, clinical characteristics and outcome.

**Results:**

The PaO2/FiO2 ratio was lower in the patients of Group A (46.8 vs 89.7 [median]) despite the PEEP values being higher (15.0 vs 8.5 [median]). The Murray score was higher in Group A (3.50 vs 2.75 [median]). During the transfer there were no significant complications noted in Group A, whereas two patients in Group B were reported with hypoxia (SpO2 < 90%). One patient in Group A died. All the other patients of the two groups have been discharged from hospital.

**Conclusions:**

The creation of an ECMO team, with various experts in the treatment of ARDS, assured a safe transfer of patients with severe hypoxia, over long distances, when in other cases they wouldn't have been be transportable.

## Background

Extra-corporeal membrane oxygenation (ECMO) is generally used in the treatment of patients with extremely severe but potentially reversible pulmonary disorders [1,2]. The new influenza A(H1N1) pandemic affected Italy during the 2009 winter. It caused an epidemic of critical illness with a large number of patients admitted to the intensive care unit (ICU) [3,4]. A proportion of these patients presented or developed severe acute respiratory distress syndrome (ARDS)[5,6]. Several reports described the need of ECMO for treatment of refractory hypoxaemia, hypercapnia which occurred despite mechanical ventilation and rescue ARDS therapy [1,2]. The patients should ideally be transported to an ECMO centre before respiration becomes critically unstable as it is thereafter impossible to transport them by conventional means. In some severely ill patients, it is sometimes necessary to initiate ECMO at the local hospital and, thereafter, to transport the patient back to the ECMO centre. This report describes the organization of an ECMO-centre in central Italy, from triage by telephone to the phase of inter-hospital transportation with ECMO, describing techniques and equipment used. We also compared the safety of transport and the outcome of patients with H1N1-related ARDS on ECMO, transferred from peripheral hospitals, with patients who were transferred to our centre by conventional means.

## Methods

The Italian Ministry of Health identified 11 ECMO centres to which critically ill patients with H1N1-induced respiratory failure could be transferred, thereafter it developed criteria for the evaluation of ECMO need in patients admitted to peripheral hospitals. Following the Italian Ministry of Health dispositions, the Intensive Care Unit (ICU) of the Emergency Department of a tertiary trauma centre (Careggi Teaching Hospital, Florence, Italy) became one of the referral centres for Central Italy for H1N1-induced ARDS. Up to 31 January 2010 the H1N1 cases requiring respiratory support in Italy were 443, and twelve (2.7%) were treated in our ECMO Centre [7].

Table 1 summarizes criteria following Ministerial Guidelines, for ECMO-need evaluation in patients admitted to peripheral hospitals. From September 2009 to January 2010 our ECMO-centre received reports of 18 patients with H1N1-induced ARDS from other hospitals. Six patients had contraindications to treatment with ECMO and remained in the local hospital: three patients were terminally ill with a short life expectancy, two patients with advanced and prolonged multiple organ dysfunction, one patient with severe chronic lung disease. Twelve patients were transported to our centre and were included in this study. Four patients were transported on ECMO and eight with conventional transport. The patients were divided into 2 groups (Group A: 4 patients transferred on ECMO; Group B: 8 patients transferred with conventional transport). All patients included in this study had an H1N1 infection confirmed by real-time reverse transcriptase-polymerase-chain-reaction (RT-PCR) assayed on pharyngeal swab or bronchial lavage. Not included were the patients with clinical suspicion of H1N1 infection without RT-PCR confirmation. Informed consent was obtained from patients or their relatives. This study, supported by institutional funds only, followed the principles of the Helsinki declaration and was approved by the Internal Review Board.

**Table 1:** Contact criteria to discuss the need of ECMO

	Acute respiratory failure with 1 of the following condition:
	

1.	SaO2 < 85% for at least 1 hour

2.	Oxygenation Index >25 for at least 6 hours after ventilation's optimisation

3.	PaO2/FiO2 < 100 with PEEP ≥ 10cmH2O for at least 6 hours after ventilation's optimization
4.	Hypercapnia with pH < 7.25

5.	SvO2 < 65% with hematocrit > 30 and under vasoactive drugs infusion

The Italian Ministry of Health dispositions for first contact criteria to discuss the need of ECMO in patients admitted to peripheral hospitals.

The parameter are referred to a condition of lung protective ventilation's (tidal volume:4-6 ml/Kg of predicted body weight; plateau pressure ≤ 30 cmH20; PEEP >lower inflection point of the curve pressure-volume).

PEEP: positive end-expiratory pressure; PaO2: arterial oxygen partial pressure; FiO2: inspired oxygen fraction; RR: respiratory rate; SaO2: peripheral oxygen saturation; Oxygenation Index: Mean airway pressure (cmH2O) * FiO2 * 100/PaO2; SvO2: central venous oxygen saturation.

### Organization of the ECMO-Centre

The ECMO service was activated by phone call from the peripheral hospital, and the intensivist on duty submitted a questionnaire to evaluate the indication for ECMO treatment. When possible, the patient was transferred by conventional transport to the ECMO Centre. If the clinical condition was unstable and/or the local hospital too far to assure safe transport, the ECMO-team was activated. The ECMO-team was activated in the presence of this condition: PaO2/FiO2 < 70 or PaCO2 > 55 mmHg. The parameter refers to a condition of protective lung ventilation (tidal volume:4-6 ml/Kg of predicted body weight; plateau pressure ≤ 30 cmH20; PEEP > lower inflection point of the pressure-volume curve). The internal activation system counted on the possibility to recruit all the professional figures involved in ECMO-team in less than one hour. The health personnel involved were: intensivist, cardiac surgeon, cardiologist, perfusionist and nurses. All these figures had previously been trained in ECMO technique and management.

### Equipment and transport

An ambulance and a medical car were used for the transport of medical staff and the equipment. The ambulance was planned for this use, it has internal spaces and adapted support for the attachment of the ECMO and the electro-medical equipment in order to guarantee a safe transportation. A mobile structure had been developed for the transport of all ECMO components: circuit tubing, rotaflow pump, membrane oxygenator and oxygen tank. The structure was carried by hand without a cart and could be safely fixed to a support of the ambulance. The ECLS device was a rotaflow Maquet Centrifugal Pump (Maquet, Rastatt, Germany) connected in series to a hollow fibre membrane oxygenator (Quadrox-D Oxygenator, Maquet, Rastatt, Germany) and biocoated circuits. Additional equipment components were a mechanical ventilator (Dräger Oxylog^® ^3000), multiparametric monitor (Dräger Infinity^® ^Delta XL), defibrillator (Medtronic LIFEPAK^® ^12), oxygen tanks and three portable boxes containing material for cannulation.

### Bedside clinical evaluation

The decision to use the device was preceded by a thorough clinical assessment of the patient which included: compiling their past and recent medical history; viewing X-rays or CT (if any); determining a pressure-volume static curve with the measurement of upper and lower inflection points; lung recruitment manoeuvres; lung ultrasound examination; evaluation of cardiac function and the measurement of pulmonary pressure with trans-thoracic and trans-oesophageal echocardiography, if MPAP was > 45 mmHg we inserted a pulmonary artery catheter to monitor the pulmonary pressure.

According to our internal protocol, if there was inclusion criteria, ECMO was begun at the local hospital.

### Patient care

Prior to placement of the cannulae, vascular ultrasound examination was performed to assess the calibre and patency of the jugular and femoral vein. Cannulae were positioned percutaneously. The Seldinger technique was used and the wire was placed with vein visualization by real-time echography. The skin and fascia would be thoroughly dilated after the wire was placed. The cannulae were Maquet Cannulae (21-28 French, Maquet, Rastatt, Germany). The blood was drained from the right atrium through a cannula introduced via femoral vein and was returned to the jugular vein. A recent acquisition was the Avalon Elite™ Bi-Caval Dual Lumen Catheter (DLC): a single cannula allowed simultaneous venous drainage and re-infusion of blood via the internal jugular vein. Cannulae positioning was controlled by real-time TEE performed by a cardiologist. We used a continuous infusion of Eparin for systemic anticoagulation. Heparinization was controlled by bedside activated partial thromboplastin time (aPTT) with Hemocron (Hemochron Jr. Sign. plus, ITC Europe, Milan, IT) every two hours during transport. High flow technique (4-6 litres per minute) was adopted according to clinical conditions and cardiac output. The desired temperature was maintained by a heat-exchanger, which was connected to the oxygenator. Blood flow and fresh gas flow were usually delivered at 1:1 ratio. Ventilation was regulated on protective parameters to maintain plateau pressure lower than 25 cmH20, tidal volume equal to 4 ml per Kg of predicted body weight and the inspired oxygen fraction, lower than 0.5. PEEP, was established at 2 cmH2O above the lower inflection point. Chest X-ray was performed prior to transfer to confirm the correct positioning of the cannulae and to exclude the possibility of a pneumothorax. When the haemodynamics and respiratory parameters stabilized, the transport to the ECMO Centre was activated. During transport the following parameters were monitored: heart rate, invasive arterial pressure, pulmonary artery pressure, peak airways pressure, peripheral oxygen saturation, flow and rpm of ECMO and PTT every two hours. The staff wore full protective garments (including FFP3 respirators, 3M Italia SpA, Segrate, Italy)

### Statistical analysis

The continuous variables are reported as medians and range.

## Results

Table 2 summarizes the clinical characteristics of patients at the moment of ECMO-centre activation and transport characteristics. The average distance from the local hospital to the ECMO-centre was similar in both the groups (78 km and 80 km (median)). All the ECMO transportation were carried out by ambulance, the conventional transport included two aeroplanes and one helicopter transfer. The ECMO-team were ready to act 165 minutes (on average) after the call and the transfer of the patient on ECMO lasted 125 minutes (on average); during the transfer no significant complications were noted. Hypoxia was reported in two patients of Group B during the transfer. The PaO2/FiO2 ratio was lower in the patients in Group A (46.8 vs 89.7(median)) despite the PEEP values being higher (15 vs 8.5 (median)). The Murray score was higher in group A (3.50 vs 2.75 (median). Table 3 contains the clinical data of the patients during their hospitalisation in the ECMO-centre, and their outcome. Three patients in Group B were treated with ECMO in our centre, two within the first 24 hours of hospitalisation and one after seven days. The duration of ECMO support was similar in two groups. In 5 patients treated with ECMO in our ICU we noted bleeding at the entry of cannulisation and from the airway but neither were fatal. Fifty-four percent of the patients contracted another infection during hospitalisation; 5 out of 7 patients treated with ECMO contracted a bacterial or fungal infection during their ICU stay. In 2 patients legionella were present in BAL cultures at the time of ICU admission, therefore the viral infection was probably a secondary infection. The other secondary infections caused by opportunistic germs were most likely the expression of VAP after an extended period of mechanical ventilation and were not related to transport. The duration of mechanical ventilation was 14.5 days in Group A compared to 10.5 days in Group B (median). The ICU-LOS was similar in the two groups (16 vs 13.5 (median)). The hospital LOS in the two groups did not differ. A patient in group B had a very long hospitalisation (hospital LOS = 107 days) owing to the presence of pleural empyema requiring several surgical operations. One patient in Group A died. The death occurred from intractable respiratory failure due to Aspergillus infection. All the other patients of the two groups were discharged from hospital.

**Table 2:** Clinical characteristics of patients in peripheral hospital

	GROUP A (N patients:4)		GROUP B (N patients:8)	
	**Median**	**Range**	**Median**	**Range**

Age (years)	44.5	30-48	43.5	15-53

BMI	36.35	24.5-56.2	27.3	19.6-27.3

Distance from the local hospital to the ECMO-centre (Km)	78	35-520	80	25-740

Transfer time (minutes)	125	25-240	40	15-240

Duration of MV (days)	2.5	1-6	1	1-3

SAPS II score	42	38-54	28	24-66

SOFA score	13.5	11-16	11	4-16

ICU LOS (days)	3	1-4	1.5	2-7

Hospital LOS (days)	3.5	1-4	2	2-7

PaO2/FiO2	46.8	45.6-54.9	89.7	66.0-116.0

PaCo2 (mmHg)	63.9	54.0-89.0	49.7	21.0-63.8

PH	7.26	7.22-7.32	7.35	7.18-7.48

Peep (cm H2O)	15	10-16	8.5	6.0-10.0

Murray score	3.50	3.25-4.00	2.75	2.25-3.00

Cst (ml/cm H2O)	29.5	13.0-40.0	37	26-58

Clinical characteristics, gas exchange of patients in peripheral hospital at the moment of ECMO-centre activation and transport characteristics. All patients were at fractional inspired concentration of oxygen (FiO2) = 1.0. PEEP: positive end-expiratory pressure; PaO2: arterial oxygen partial pressure; PaCO2: arterial carbon dioxide partial pressure; LOS: length of stay; MV: mechanical ventilation; BMI: body mass index; SAPS: Simplified Acute Physiology Score; SOFA: Sequential Organ Failure Assessment; Cst: static pulmonary compliance.

**Table 3:** Clinical data in the ECMO-center and outcome

	GROUP A (N patients:4)		GROUP B (N patients:8)	
	**Median**	**Range**	**Median**	**Range**

Duration of ECMO (days)	9.5	8-28	8	0-19

Duration of MV (days)	14.5	12-35	10.5	4-48

ICU LOS (days)	16	14-35	13.5	4-81

ICU LOS-T(days)	19	15-38	15	8-87

Hospital LOS-T (days)	39.5	31-47	27.5	6-107

ICU mortality, % (N)	25%(1)		0%(0)	

Hospital mortality, % (N)	25%(1)		0%(0)	

Clinical data of the patients during their hospitalisation in the ECMO-centre and the outcome.

LOS: length of stay; LOS-T: total length of stay (peripheral hospital+ecmo center); MV: mechanical ventilation.

## Discussion

Influenza A, H1N1 infection, can induce severe ARDS and cases of conventional ventilation failure have been reported; the transfer of these patients to specialized ARDS centres is often necessary permitting advanced treatment when ECMO is necessary. Occasionally, if the clinical case was particularly unstable and the transfer of this patient was risky using conventional transport, it was necessary to start the ECMO treatment in the local hospital and continue ECMO during the transfer. The use of a check-list during the telephone interview enabled the identification of patients treatable whose conditions were more serious, and the ECMO team was activated only for a third of the cases. In the remaining cases the transfer of the patient to the ECMO centre was carried out with conventional means.

Two patients experienced hypoxia during the transfer using conventional ventilation with SpO_2 _< 90%; this was because H1N1-induced ARDS evolves rapidly and there is often a decline in oxygenation of the blood in few hours. This was shown by the fact that the patients had been ventilated only for a few days before the call to our centre and three of the patients transported in the traditional manner were treated with ECMO in our centre, two within 24 hours of arrival, one after seven days. The Group A patients' conditions were, on average, more serious than Group B patients at the moment of evaluation in terms of PaO2, PaCO2 and PEEP; perhaps because they had previously been ventilated for a longer period than the others: 2.5 vs 1.0 days (median), the activation was tardier.

It is important to underline the fact that all patients with ECMO criteria were treated, with no exclusion from treatment independently from the seriousness of the case. Several studies noted various complications during the transfer in ECMO (bleeding, circuit occlusion, breakages in the circuit, etc.) [8-11]; in our experience, no complications during the transfer on ECMO occurred. The clinical evaluation and the advanced treatment by the ECMO team, a multidisciplined equip with expertise in ARDS and ECMO treatment, allowed the optimization of the clinical parameters and safe transport, even over long distances (520 Km), of patients with extremely serious ARDS.

The evolution of the respiratory insufficiency from H1N1 to ARDS determined a serious hypoxaemia which did not respond to treatment with high PEEP or high FiO2 and was not easily treated with extracorporeal oxygenation at low flow. The use of ECMO with medium-high flow (4-6 l/min) allowed a rapid correction of blood gases and a speedy transfer to the ECMO centre. Furthermore, the rapid resolution of the hypoxia allowed us, from the start, to carry out a protective ventilation strategy during the transport and for the duration of the hospitalization.

There was no difference in days of mechanical ventilation, ICU-LOS and hospital LOS between the two groups; one patient in Group A died, none in Group B.

Little has been written on transport while on ECMO for ARDS in adults [8,10,11]; it has been described only in one case report for ARDS-related H1N1 [12]. A recent study described approximately 200 patients with H1N1-related ARDS treated in 15 Australian and New Zealand ICUs which used ECMO treatment [13], the population described is extremely similar to ours in terms of age, BMI and comorbidity. In this study 30% of patients (53) against 50% in our centre (7) were treated with ECMO. This result is explained by the fact that as our centre is the point of reference for many regions in central Italy, we treated the more serious patients; patients who were less serious were treated in the ICU of their local hospital. The 53 patients treated with ECMO were similar to our 7 patients in seriousness; even the duration of the ECMO treatment by mechanical ventilation, ICU-LOS and Hosp. LOS were similar. In this study the mortality rate was of approximately 15% (23% treated with ECMO, 13% not treated with ECMO) against our 7%. In our centre we have not registered deaths of patients on mechanical ventilation. This difference is partly explained by the fact that the patients centralized in our ICU were only those potentially curable with ECMO without criteria of exclusion, the patients with criteria of exclusion from ECMO treatement stayed at the local hospitals.

The limitation of our study is the impossibility to compare the mortality rate of patients treated with ECMO with other reports because the sample was too small.

## Conclusions

The A H1N1 influenza can induce serious ARDS and non-conventional treatment like ECMO, can be safely instituted in highly specialized centres. Due to a rapid progression of respiratory failure, transfer of these patients to a referral centre on mechanical ventilation is often infeasible. Therefore the start of ECMO treatment at the peripheral hospitals and transportation on ECMO could be a viable option. The creation of an ECMO team, with experts in the treatment of ARDS, seems a key aspect for the safe management of these patients, including when the most advanced technique are not immediately available.

## Declaration of interests

The authors declare that they have no competing interests.

## Authors' contributions

MC, GC, GZ have made a substantial contributions to design of the study. SB, AP, RS, CG, MB, MB, CL, PB, AP have made a substantial contributions to acquisition, analysis and interpretation of data. All authors read and approved the final manuscript

## References

[B1] Schuerer DJ Kolovos NS Boyd KV Coopersmith CM Extracorporeal membrane oxygenation: current clinical practice, coding, and reimbursement Chest 2008 134 179 184 10.1378/chest.07-2512 18628221

[B2] Peek GJ Mugford M Tiruvoipati R Wilson A Allen E Thalanany MM Hibbert CL Truesdale A Clemens F Cooper N Firmin RK Elbourne D Efficacy and economic assessment of conventional ventilatory support versus extracorporeal membrane oxygenation for severe adult respiratory failure (CESAR): a multicentre randomised controlled trial Lancet 2009 374 1351 1363 10.1016/S0140-6736(09)61069-2 19762075

[B3] Perez-Padilla R de la Rosa-Zamboni D Ponce de Leon S Hernandez M Quinones-Falconi F Bautista E Ramirez-Venegas A Rojas-Serrano J Ormsby CE Corrales A Higuera A Mondragon E Cordova-Villalobos JA Pneumonia and respiratory failure from swine-origin influenza A (H1N1) in Mexico N Engl J Med 2009 361 680 689 10.1056/NEJMoa0904252 19564631

[B4] Jain S Kamimoto L Bramley AM Schmitz AM Benoit SR Louie J Sugerman DE Druckenmiller JK Ritger KA Chugh R Jasuja S Deutscher M Chen S Walker JD Duchin JS Lett S Soliva S Wells EV Swerdlow D Uyeki TM Fiore AE Olsen SJ Fry AM Bridges CB Finelli L Hospitalized patients with 2009 H1N1 influenza in the United States, April-June 2009 N Engl J Med 2009 361 1935 1944 10.1056/NEJMoa0906695 19815859

[B5] Rello J Rodríguez A Ibañez P Socias L Cebrian J Marques A Guerrero J Ruiz-Santana S Marquez E Del Nogal-Saez F Alvarez-Lerma F Martínez S Ferrer M Avellanas M Granada R Maraví-Poma E Albert P Sierra R Vidaur L Ortiz P Prieto del Portillo I Galván B León-Gil C Intensive care adult patients with severe respiratory failure caused by Influenza A (H1N1)v in Spain Crit Care 2009 13 R148 10.1186/cc8044 19747383 PMC2784367

[B6] Chowell G Bertozzi SM Colchero MA Lopez-Gatell H Alpuche-Aranda C Hernandez M Miller MA Severe respiratory disease concurrent with the circulation of H1N1 influenza N Engl J Med 2009 361 674 679 10.1056/NEJMoa0904023 19564633

[B7] Nuova influenza http://www.nuovainfluenza.salute.gov.it

[B8] Lindén V Palmér K Reinhard J Westman R Ehrén H Granholm T Frenckner B Inter-hospital transportation of patients with severe acute respiratory failure on extracorporeal membrane oxygenation-national and international experience Intensive Care Med 2001 27 1643 1648 10.1007/s001340101060 11685306

[B9] Zimmermann M Bein T Philipp A Ittner K Foltan M Drescher J Weber F Schmid FX Interhospital transportation of patients with severe lung failure on pumpless extracorporeal lung assist British Journal of Anaesthesia 2006 96 63 66 16299045 10.1093/bja/aei274

[B10] Wagner K Sangolt GK Risnes I Karlsen HM Nilsen JE Strand T Stenseth LB Svennevig JL Transportation of critically ill patients on extracorporeal membrane oxygenation Perfusion 2008 23 101 106 10.1177/0267659108096261 18840578

[B11] Haneya A Philipp A Foltan M Mueller T Camboni D Rupprecht L Puehler T Hirt S Hilker M Kobuch R Schmid C Arlt M Extracorporeal circulatory systems in the interhospital transfer of critically ill patients: experience of a single institution Ann Saudi Med 2009 29 2 110 4 10.4103/0256-4947.51792 19318758 PMC2813631

[B12] Grasselli G Foti G Patroniti N Giuffrida A Cortinovis B Zanella A Pagni F Mergoni M Pesci A Pesenti A A case of ARDS associated with influenza A-H1N1 infection treated with extracorporeal respiratory support Minerva Anestesiologica 2009 75 N.12 741 745 19940827

[B13] Davies A Jones D Bailey M Beca J Bellomo R Blackwell N Forrest P Gattas D Granger E Herkes R Jackson A McGuinness S Nair P Pellegrino V Pettilä V Plunkett B Pye R Torzillo P Webb S Wilson M Ziegenfuss M Extracorporeal Membrane Oxygenation for 2009 Influenza A(H1N1) Acute Respiratory Distress Syndrome Jama 2009 302 1888 1895 19822628 10.1001/jama.2009.1535

